# Design and application of synthetic human gut microbial communities

**DOI:** 10.1080/19490976.2025.2575923

**Published:** 2025-11-15

**Authors:** Min Soo Kim, Jordan E Bisanz

**Affiliations:** a Department of Biochemistry and Molecular Biology, The Pennsylvania State University, University Park, PA, USA; b One Health Microbiome Center, Huck Institutes of the Life Sciences, The Pennsylvania State University, University Park, PA, USA

**Keywords:** Microbiome, dysbiosis, synthetic community, defined microbial consortium, live biotherapeutic products, infectious disease, colonization resistance, inflammable bowel disease, cancer, immunotherapy

## Abstract

The gut microbiome shapes host health through a complex network driven by both host‒microbe and microbe‒microbe interactions. Disruption of these interactions, often referred to as dysbiosis, is associated with a range of infectious and chronic diseases. Owing to the success of fecal microbiota transplantation (FMT) for the treatment of recurrent *Clostridioides difficile* infection, FMT has been explored as a therapeutic option for a range of microbiota-associated conditions, including inflammatory bowel disease and obesity. However, the microbial diversity that is the greatest strength of FMT is also its greatest liability. Concerns relating to reliance on human donors, potential for transmission of multidrug-resistant organisms or undesirable phenotypes demonstrate a need for alternate approaches, including the generation of synthetic alternatives to FMT, which can be built in the laboratory from individual strains. Furthermore, these communities are powerful tools for conducting mechanistic research allowing for the generation of ‘knockout’ communities, which are not possible when working with undefined fecal transplants. This review examines strategies for designing synthetic microbial communities that represent a new generation of microbiome-derived therapies. We highlight how synthetic microbial communities are being used to answer mechanistic questions about host–microbiome interactions relevant to health and disease. Finally, we examine the current clinical translation of these communities as live biotherapeutic products (LBPs). While the regulatory frameworks for LBPs continue to evolve, early clinical successes illuminate the potential for synthetic microbial communities to treat complex human diseases through targeted manipulation and restoration of the gut microbiome.

## Introduction

In the human gastrointestinal tract, trillions of microbes including bacteria, fungi, archaea, and viruses reside to form a community, the microbiota, which shapes the health and physiology of the host.^1–3^ In particular, bacteria in the microbiota contain the capacity to produce small molecules, peptides, and proteins that mediate countless microbe-microbe and microbe-host interactions critical to maintaining homeostasis.^4^ Some of these metabolic outputs can not only directly interface with the intestinal environment, but also enter circulation and influence biological processes at distal sites including the liver, brain, and lung.^5^ Disruption of the gut microbiome (dysbiosis) has been linked to numerous adverse clinical outcomes, such as metabolic^1^^,^^6^^,^^7^ and immune disorders,^8^^,^^9^ infections,^10^^,^^11^ and cancer.^12^^,^^13^

Realization of the clinical relevance of enteric symbionts coincided with rapid advancements in sequencing and analytical technologies, leading to an explosion of metagenomic data and mechanistic insights behind key microbe-microbe and microbe-host interactions.^14-16^ While significant progress has been made in recent decades to decouple the relationship between microbial ecology in the gut and its impact on the host, the sheer complexity of the gut microbiome remains a substantial obstacle in establishing causality and developing effective targeted therapeutic strategies. In the absence of more precise approaches, therapeutic administration of healthy donor fecal material, fecal microbiota transplantation (FMT), has reemerged as a viable tool.^17^ The practice of FMT is thought to date back to 4th century China, where it was termed “yellow-soup”. However, its modern resurgence began in the early 2010s as a therapy for recurrent *Clostridioides difficile* infection (rCDI).^18^ rCDI offers an attractive first target for FMT, as conventional antibiotic therapies exacerbate the underlying dysbiosis, which leads to the initial *C. difficile* infection, and predisposes patients to reinfection.^19^ Since the adoption of this practice, numerous pieces of evidence from randomized controlled trials, systematic reviews, and meta-analyses have corroborated the clinical benefits of FMT in preventing the recurrence of CDI.^20–23^ Despite the efficacy of FMT for treating rCDI, severe adverse events (SAEs), including death, have been reported following FMT.^24^^,^^25^ Most SAEs have been associated with the transmission of infectious agents, including multidrug-resistant organisms and both enteropathogenic and Shiga toxin-producing *E. coli*. These outcomes were attributed to inadequate donor screening and highlighted the need for strict guidelines for screening donor material. Taken together, these observations highlight the inherent safety challenges that accompany the use of undefined microbial communities.^26^

An alternative approach is to replace FMT with a synthetic product that can be built under controlled conditions and be composed of purified strains with favorable safety profiles allowing for reproducibility and predictability. While a simple task in theory, one major knowledge gap has precluded the generation of these microbiome-derived therapeutics: which microbial strains need to be included for clinical efficacy? The gut microbiota contains thousands of species,^27^ and intraspecies/infraspecific genetic variation adds exponential complexity to the question.^28^

This review describes current approaches to the design and validation of synthetic microbial communities to answer questions about how gut microbes shape host health and how they are being used in clinical practice. We also discuss the emergence of defined microbial consortia as a category of live biotherapeutic products (LBPs) to treat a myriad of diseases and the opportunities they present for the future of personalized and precision medicine.

### Synthetic community design strategies

A common schema in microbiome research is to conduct cross-sectional analyses from healthy and diseased participants, combined with sequencing or other omics approaches to identify differentially abundant features that may represent strains, genes, or metabolic pathways. While this approach is often effective in establishing correlative relationships between the microbiota and health, a renewed focus has been placed on replicating these phenotypes *in vivo* using fecal transplants in gnotobiotic or antibiotic-treated animal models to provide evidence of a causative role.^14^^,^^29-32^ However, the lack of experimental tractability for complex fecal samples and the inability to faithfully recapitulate the community structure in mice limits further mechanistic investigations.^33^ To address this challenge and provide a more complete understanding of how the microbiome impacts the host, a body of work has emerged to develop defined synthetic microbial communities, commonly referred to as SynComs, to be utilized in lieu of raw human fecal material. In this review, we delineate synthetic communities from conventional multistrain probiotics, in that multistrain probiotics are generally composed of FDA-approved generally recognized as safe (GRAS) lactobacilli, bifidobacteria, and bacilli, which are commonly found as over-the-counter supplements. Furthermore, both the terms community and consortia are commonly used in the field to describe these synthetically constructed communities. In this review, we favor the term community because it better enforces the cooperative nature of the group of microbes designed to perform specific functions.

Among the first examples of the synthetic community approach, Schaedler et al. developed a defined microbial community modeling the mouse gut microbiota for experiments in germ-free (GF) mice in 1965.^34^^,^^35^ This work was motivated by the physiological abnormalities observed in GF animals, including a greatly enlarged cecum, which was shown to be reversible by colonization.^36^^,^^37^ The 6-member Schaedler flora (SF) was able to successfully colonize the murine gut within a week of introduction and persist in the gastrointestinal tract for several months while restoring normal cecum size. While the SF was used for subsequent gnotobiotic studies, it mostly contained aerotolerant and facultative anaerobes for logistical reasons and lacked representation of the strict anaerobes that reside in the murine gut. In 1978, Orcutt et al. developed an 8-member variant termed Altered Schaedler flora (ASF) that contains four members of the SF and four new species, including three strict anaerobes, selected for their abundance in the murine gut, ease of cultivation, and identification by microscopy.^38^^,^^39^ Since its development, ASF has continued to serve as a reference microbiome and minimally defined community in many studies investigating the gut microbiota in mice. While conceptually innovative, questions remain as to the extent to which ASF truly models the typical composition of the mouse gut microbiota and its translational relevance to the human gastrointestinal tract.

**Figure 1.**
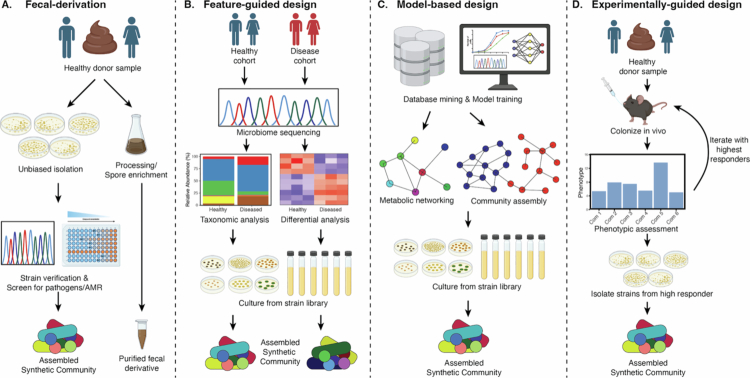
Synthetic Community Design Strategies. (A) In the fecal derivation approach, stool samples from healthy donors are cultured to isolate as many strains as possible. Cultured strains are sequenced to verify their identity and are often screened for antimicrobial resistance. All or most unique strains are pooled to form a synthetic community. Alternatively, a healthy donor sample could undergo processing, such as ethanol- or chloroform-based purification, to enrich target populations, such as spore-forming bacteria. (B) In feature-guided design, sequences or other ‘omics features are identified through approaches including differential abundance analysis in cross-sectional cohort designs. The features predicting healthy states can be identified in cultured isolates and reconstituted together to form a synthetic community. (C) Model-guided design leverages computational tools to predict optimal strain combinations based on their metabolic capacity, fitness, and gene content. (D) Experimentally guided approaches aim to identify strains that lead to specific beneficial phenotypes through iterative and/or permutational community design to optimize strain membership.

In the past two decades, there has been enormous growth in both metagenomic and reference genome databases owing to major efforts, such as the Human Microbiome Project,^14^^,^^15^^,^^40^^,^^41^ Broad OpenBiome,^42^ PRISM,^43^ and the development of many computational tools to extract and profile the microbial constituents at varying levels of resolution.^32^^,^^44^^,^^45^ It is increasingly possible to mine the human gut microbiota for species, both known and unknown, with greater taxonomic and functional specificity, allowing for the expansion of strategies to design, develop, and manufacture synthetic communities. Broadly speaking, synthetic communities may be designed through both top-down and bottom-up approaches, but these definitions do not fully capture the conceptual approaches that underlie the development of synthetic communities for both mechanistic and therapeutic approaches. In this review, we describe four primary approaches that guide the development of synthetic communities. These strategies are neither exhaustive nor exclusive, but they serve as a framework to contextualize the underlying approaches for rational community design. These approaches are (i) fecal derivation, (ii) feature guided, (iii) model based, and (iv) experimentally guided (Figure 1). These approaches are often complementary, with many studies employing combinations of design strategies. While many defined microbial communities have been developed over the years to investigate microbial ecology across environmental niches, we will focus on synthetic communities developed to study clinically relevant phenotypes related to the human gut microbiota (Table 1).

**Table 1.** Highlighted synthetic communities in basic science research.

Name	Number of species	Approach	Disease Model	References
RePOOPulate/MET−1	33	Fecal derivation	CDI	Petrof et al.[46]
			*S.* Typhimurium infection	Martz et al.[47]
17-mix	17	Experimentally guided	Immune modulation	Atarashiet al.[48]
-	94 consortia of diverse sizes	Model-based	Immune modulation	Faith et al*.*[49]
O-MM12	12	Fecal derivation	*S.* Typhimurium infection	Brugiroux et al*.*[50]
O-MM12 + FA3	15			
BCT	10	Fecal derivation	IBD	Li et al*.*[51]
SIHUMI	7	Fecal derivation	Immune modulation	Becker et al.,[52] Ring et al*.*[53]
SIHUMIx	8			
-	6	Feature-guided	Food Allergy	Abdel-Gadir et al*.*[54]
11-mix	11	Experimentally-guided	Cancer Immunity	Tanoue et al*.*[55]
GUT−103	17	Experimentally-guided	IBD	van der Lelie et al*.*[56]
GUT−108	11			
hCom2	119	Feature-guided	EHEC infection	Cheng et al*.*[57]
B10	10	Feature-guided	CDI	Liu et al.[58]
SC−4	4	-	IBD	Ambat et al*.*[59]
sFMT	37	Feature-guided	CDI	Tian et al*.*[60]
SynCom	18	Model-based	*F. nucleatum-mediated CRC*	Zhou et al.[61]
DefCom1	24	Fecal derivation	CDI	Fishbein et al*.*[62]
DefCom2	48			

#### Fecal derivation

The hallmark characteristic of this approach is that communities are not necessarily “designed” in the true sense but rather are purified, or semi-purified, from donor material (Figure 1A). These approaches constitute some of the first wave of human gut synthetic community approaches harnessing the innate complexity of the human microbiome.

The semi-purified approach is best typified by SER−109, described later under clinical applications. These communities are enriched for organisms of interest through a variety of methods. SER−109 competes with *C. difficile* through the chemical enrichment of spores*.* This spore enrichment approach aids in product stability while neutralizing many potential opportunistic pathogens.

Alternatively, in the purified approach, which generates a true synthetic community, extensive culture of donor fecal material is performed to isolate the most abundant and prevalent culturable microbes found in the sample. These isolated strains are rigorously characterized through sequencing and phenotyping to identify potential safety concerns, such as antimicrobial resistance and virulence factors. All strains passing these tests can then be pooled back together to form the synthetic community. As these strains naturally coexist within the donor, they may be expected to form a stable community without excluding each other in the recipient, representing a unique conceptual advantage of this approach. This is an important consideration, as competition among strains often leads to exclusion or suppression, which has been well demonstrated in the common gut microbe *Bacteroides* spp.^63-65^ One of the earliest examples of this fecal-derivative approach is RePOOPulate.^46^ RePOOPulate was developed by untargeted culture of strains from the fecal material of a healthy female donor. A total of 62 strains were isolated, and after screening for antimicrobial resistance, 33 strains were selected for the final community. This community was administered to two patients with rCDI, with both recipients demonstrating evidence of engraftment and clinical efficacy, including no detectable *C. difficile* toxin 24−26 weeks after administration despite both receiving additional antibiotic administration owing to unrelated complications. To our knowledge, this is the first successful example of the synthetic community approach being applied for therapeutic purposes in humans, setting the stage for the development of microbial therapeutics that is distinct from conventional multistrain probiotics.

#### Feature-guided design

Given that fecal-derivation approaches are guided by enrichment or are based on the selection of highly abundant and easily cultured strains, these approaches inherently assume that all the microbes formulated are beneficial, which may or may not be true. In the case of *C. difficile* infection, organisms such as *Clostridium scidens*, *Dorea* spp., and *Peptostreptococcus* spp.^60^^,^^66^^,^^67^ may be beneficial, while other commensals, such as *Enterococcus* spp.^68^ may be detrimental. While there are described mechanisms in the cases above, in many contexts, this information is incomplete. As an alternative, feature-guided community design (Figure 1B) leverages high-throughput sequencing or other ‘omics approaches combined with *in silico* tools to identify putative therapeutic strains or metabolic pathway-containing strains. In the simplest sense, this may involve the identification of strains through differential abundance analysis between healthy and disease cohorts. Feature-guided design poses 3 distinct challenges: (i) matching sequence features to isolates, (ii) culturability and stability of target strains, and (iii) incompatibility between target strains.

Identification of taxa through sequencing is generally accomplished through 16S rRNA gene sequencing or metagenomic sequencing. While these approaches have differing levels of resolution, both present a fundamental challenge of translating genetic sequences to physically culturable organisms. In the most straightforward approach, sequences may be assigned a taxonomic identification through a database, and a matching isolate could be identified. This approach is highly dependent on database selection, and the shifting standing nomenclature of many common gut microbes may render this approach challenging. Furthermore, the genetic and phenotypic variation among strains of the same species, as exemplified by pathogenic vs probiotic *E. coli* suggests that this coarse level of resolution may not be appropriate in many contexts.^28^ Preferably, comparisons between sequence features and strain databases should be made quantitatively. In 16S rRNA gene sequencing, this may be implemented through percent identity requiring a matching strain to have >97% identity favoring those strains with the highest identity. In metagenomic sequencing, this task may involve the assembly of metagenome-assembled genomes (MAGs) and subsequent whole-genome average nucleotide identity where >94−95% may represent the same species and >99.9% represents a strain.^69^ With access to original samples, it is possible to isolate microbes on the basis of perfect matches between an isolated strain and metagenomic sequences.^70^^,^^71^ However, this method scales poorly to a large number of strains, particularly those without well-established selective and differential media.

While there are qualitative and quantitative definitions of a bacterial species, the term strain does not carry a precise definition when considered in the context of metagenomes. In this review, we define a strain as an approximately clonal population that can be isolated and propagated in pure culture. A fundamental challenge in this definition is that the number of strains of a species is pseudoinfinite, with constant mutation and gene gain/loss events creating a moving target. As such, some level of fuzzy matching is required in these approaches, with future efforts needed to define functional thresholds for the selection of strains.

Alternatively, the functional unit of assembly need not be the strain but rather genes, metabolic pathways, or metabolites. The coverage of identified gene families and pathways could be optimized in a minimal set of microbes, but it may be desirable to encode functional redundancy by including multiple strains with the same or homologous genetic determinants to avoid community collapse from the loss of any one strain.^72-74^ Furthermore, it may be important to include strains with complementary auxotrophies to reduce the likelihood of exclusion by competition.^75^ One conceptual challenge to this approach is that a strain with a desired function may or may not contain many other detrimental functions, as even “bad microbes” may have good traits and vice versa. When a limited number of metabolic functions are required, engineered strains could be considered as alternatives.

Regardless of the approach used for matching features to isolates, both culturability and competition between strains pose an issue. Many members of the gut microbiota have yet to be cultured or may function as epibionts requiring a second organism present to grow.^76^^,^^77^ In addition, despite the policies and recommendations of scientific journals and funding agencies to share data and microbial resources, the rate of deposition of isolated strains into public collections is low owing to regulatory concerns, intellectual property priorities, and lack of enforcement.^78^^,^^79^ This introduces significant barriers to acquiring many target strains identified by their sequences. Functionally, these strains do not lend themselves to formulations in synthetic communities and can be excluded for pragmatic reasons. Furthermore, owing to the sheer complexity of microbe‒microbe interactions, not all strains are compatible with each other or with endogenous strains in the host/patient. This can be observed both in animal and human studies as variable engraftment.^60^^,^^80^ Despite these challenges, feature-based design offers an important strength in that it does not rely on prior mechanistic knowledge. This also allows feature-designed communities to serve as a platform for mechanistic study.

To illustrate this approach, we leveraged a feature-based design strategy involving machine learning to design synthetic communities.^60^ Through a meta-analysis of 12 human studies totaling 899 samples, we identified 200 microbial signatures that could robustly predict *C. difficile* colonization status and reconstructed networks of organisms anticorrelated with the CDI using proportionality analysis. The final community (sFMT1) contained 37 strains representing approximately 49.6% of the target features that predict the exclusion of *C. difficile*. Through extensive characterization of sFMT1 using multi-omics approaches, we demonstrated that strains capable of Stickland fermentation of proline were both necessary and sufficient for *C. difficile* inhibition, providing important mechanistic insights into how the healthy gut microbiota suppresses *C. difficile*, and showcasing the importance of feature-based design in hypothesis-drivien microbiome sciences.

#### Model-based design

While feature-based design allows for new mechanistic discovery, this requires significant experimental effort. As the body of literature on the individual members of the gut microbiome grows, so does the foundation of knowledge that can be leveraged to build computational frameworks to generate effective synthetic communities. The model-based design approach (Figure 1C) aims to predict optimal strain combinations based on their innate capabilities, such as metabolic activity and growth rate under physiological conditions. Many complex models have been developed to study the ecological drivers of community dynamics in the gut,^81-85^ and metabolic functions have emerged as the key factor in the stable coexistence of microbes and microbiome assembly.^86^^,^^87^ As such, many model-based approaches to build communities focus on identifying bacterial strains with synergistic metabolic pathways, complementary nutritional requirements, and cross-feeding capabilities to construct an effective robust community directed to produce specific beneficial metabolites and/or exclude pathogens.

Understanding ecosystem stability and community dynamics are key prerequisites that underpin the design of synthetic communities with targeted functions. Population dynamics models, such as the generalized Lotka-Volterra model (gLV), which captures the temporal change in species abundance using individual strain growth parameters and interspecies interactions, have been explored to predict community assembly *in silico*^88^. While gLV models usually track absolute abundances or population densities, parameterizing the models with relative abundance data commonly acquired through microbiome sequencing was found to be sufficient for estimating the key interactions and community dynamics in microbiome studies.^89^ A major advantage of the model-based approach is the ability to create and test a large number of communities to derive insight into the mechanisms behind community functions in ways that would be prohibitively labor intensive through experimental approaches.

Clark et al. demonstrated the effective use of this model-based approach to design synthetic communities with optimized functions for butyrate production using a modeling framework.^90^ They employed gLV models that predict community assembly and supplemented it with a linear regression model including interaction terms that mapped the community composition to butyrate production. Through iterative cycles of design-test-learn, the model simulated millions of communities to gain insights into organisms with the greatest impact on targeted functions, which would be impossible to complete experimentally. They successfully predicted communities that can output varying levels of butyrate and were able to identify key strains that have positive or negative impacts on butyrate production. Importantly, the model showed that the diversity of the microbiome above a threshold can constrain the maximum possible butyrate production, highlighting that communities should be designed within an optimal range of strain richness.

#### Experimentally guided design

While both feature- and model-based approaches are powerful design tools, they may not always translate to an observable phenotype or clinical efficacy. Experimental-guided design (Figure 1D) instead uses iterative and permutational screening approaches to identify functional microbes and communities that elicit phenotypes such as beneficial host responses. In a common approach for gut microbes, germ-free gnotobiotic animals may be used as a screening platform, although many disease models or *in vitro* assays could be leveraged depending on the biological question.

In one aspect of experimentally guided design involving iterative selection, animals are colonized with complex microbial communities, either designed or from complex donor material. These colonized animals may then be systematically screened for desired phenotypic outcomes. Intestinal material may then be collected from animals with the strongest responses and further administered to a new set of animals in an iterative process to enrich the microbes most strongly associated with the target phenotype. After enrichment, these communities can be characterized by sequencing, isolated, and assembled into synthetic communities. While this strategy is resource intensive, it effectively selects for functionally relevant strains with experimentally validated phenotypes. A limitation of this approach when conducted using *in vitro* or animal models is that these phenotypes may not be transferred to the context of the human gut.

As an illustration utilizing an iterative approach, Atarashi et al. developed communities to suppress inflammation in mouse models of colitis.^48^ Using elements of fecal derivation, dilutions of chloroform-treated human fecal material were transplanted into gnotobiotic mice, and the proportions of T_H_17 and T regulatory (T_reg_) cells were measured as the colitis-relevant phenotype given their significance in mediating colitis-related inflammation. From the animals that exhibited a response, 23 strains were isolated and mapped to sequencing features. Of these 23 strains, only 17 were capable of recolonizing the animals. This 17-strain mixture was capable of robustly inducing T_reg_ cells, and further reductions in complexity resulted in reductions in activity. This 17-strain mixture demonstrated clinically relevant suppression of both ovalbumin(OVA)-induced allergic diarrhea and trinitrobenzenesulfonic acid (TNBS)-induced colitis in conventional animals.

An alternative variation of experimentally guided design leverages permutational selection to generate random combinations of strains combined with meaningful phenotypic outcomes. As a formative example, Faith et al. illustrated an unbiased method for permutational analysis of synthetic communities, again focusing on multiple phenotypes, including microbial metabolite production, host adiposity, and induction of colonic T_reg_ cells.^49^ They first cultured 17 unique strains representing the four major gut phyla from a healthy female donor, which they showed to modulate T_reg_ cells. As testing all permutations of 17 strains is not feasible or ethical, they instead used a combination of *in silico* simulations and a subset of 94 communities for screening in gnotobiotic animal models. Next, with elements of model-based design, the authors used measured phenotypes and community metrics, including size and composition, to identify strains whose influence on host phenotypes was robust to larger community composition. This model-based approach could not only identify organisms that can generate functionally assembled communities but also distinguish additive microbial metabolic interactions, in which multiple strains are involved in the generation of specific metabolites or the modulation of the host immune system. Subsequent validation experiments revealed that a minimum of two strains were sufficient to induce equivalent T_reg_ induction compared to the 17-member community and that induction was not conserved among taxonomically related strains.

### Synthetic communities in basic and preclinical research

Synthetic microbial communities represent both new classes of microbiota-based therapeutics and platforms for conducting mechanistic research. In this section, we highlight selected examples of how synthetic communities are being formulated to study the mechanisms through which the gut microbiota assembles to shape host health across a variety of indications. Synthetic communities are helping to bridge the gap between correlation and causation as the microbiome sciences transition from descriptive to mechanistic research.

#### Community assembly and dynamics

Driven by a motivation to develop complex synthetic communities as a model of healthy gut microbiota, Cheng et al., ^57^ used a feature-guided approach towards the development of synthetic communities with over 100 strains present. On the basis of data derived from the Human Microbiome Project, the authors sought to identify a set of available strains that were highly prevalent in HMP participants and could maximize the mapping of their metagenomic reads onto reference genomes. After ranking features on prevalence, they identified 166 features that were present in >45% of HMP participants, of which 99 individual isolated strains could be obtained. These strains were supplemented with 5 additional strains to construct hCom1. Through *in vitro* growth experiments in defined growth media, the authors mapped the effects of varying amino acid availability on community composition, capturing results previously reported in monocultures, as well as new observations demonstrating the existence of “keystone” nutrients that determine community composition. Colonization of gnotobiotic mice with hCom1 and subsequent challenge with human fecal samples resulted in a more complex variant termed hCom2 composed of 119 members, which the authors reported comprised 50% of the estimated species in the typical human gut. This hCom2 was resilient against subsequent challenges with human fecal communities. Finally, despite the diverse and complex nature of hCom2, it was able to robustly and reproducibly colonize the murine gut and confer colonization resistance against additional human fecal matter challenge and enterohemorrhagic *E. coli* infection. This same community has further been applied to investigate how the exclusion of microbes shapes community composition and metabolism^91^ and reveal conserved T cell responses to gut commensals.^92^

#### Insights into pathogen exclusion and infection

Commensal gut microbes act as the first barrier to infection for many intestinal infections. Two common examples are *Salmonella* spp. and *C. difficile*. RePOOPulate, later termed MET−1, was developed for *C. difficile* infection as previously described. However, it has also shown efficacy in countering infection with *Salmonella* Typhimurium in murine models.^47^ Mice that received MET−1 administration displayed reduced diarrheal weight loss and an ameliorated systemic response after infection with *S.* Typhimurium. While MET−1 failed to decrease the abundance of *S.* Typhimurium in the intestines, it preserved tight junctions between enterocytes and inhibited pathogen infiltration into the systemic circulation. In another demonstration, Brugiroux et al. developed a 12-member minimal defined consortia (Oligo-MM12) that protects against *S.* Typhimurium infection in mice.^50^ Oligo-MM12 was designed by culturing the intestinal contents of specific pathogen-free (SPF) mice on nonselective media, and 12 strains representing the most abundant phyla of the murine gut were selected for inclusion. This minimal community could robustly and stably colonize the murine intestine over several generations, effectively conferring resistance to the proliferation of *S.* Typhimurium. In addition, they supplemented Oligo-MM12 with three additional facultative anaerobic strains (FA3), as they hypothesized that bacterial respiration could promote colonization resistance against *S.* Typhimurium via competition for oxygen or anaerobic electron acceptors. Indeed, Oligo-MM12 + FA3 strongly inhibited *S.* Typhimurium growth in the murine gut compared to Oligo-MM12 by itself even one day post infection and at levels comparable to those of a conventional mouse microbiota.

While exclusion may be preferable, gut microbes also modulate the virulence of many pathogens. Using donor samples from patients with varying degrees of *C. difficile* infection, Fishbein et al. identified both strains of *C. difficile* with varying degrees of virulence, and human microbial communities that can form a stable equilibrium between commensal microbes and pathogens.^62^ They assembled DefCom1 by isolating the 24 most abundant strains from the donor sample, resulting in intermediate diarrheal weight loss and no lethality in mice. In addition, to model *C. difficile*-colonized patients more broadly, they supplemented 21 strains from DefCom1 with additional taxa to represent the most prevalent genera among the entire donor population to form the 46-member DefCom2. When mice colonized with DefCom1 or DefCom2 were infected with strains of *C. difficile* with different virulence profiles, DefCom2 could more effectively suppress toxin production, and its hosts were less susceptible to all strains as measured by weight loss, despite the *C. difficile* strain-specific virulence being observed across both communities. It was hypothesized that the greater diversity of DefCom2 led to increases in the transcription of genes to modify amino acid availability for *C. difficile*, allowing the community to overcome its virulence while enabling pathogen colonization.

#### Microbial manipulation of inflammatory responses

Given the mounting evidence that microbial communities are essential for immune homeostasis in the gut, the microbiome has been studied as a potential therapeutic avenue for treatment and prevention of inflammatory bowel diseases (IBD).^43^^,^^93^^,^^94^ Bacterial consortium transplantation (BCT), a 10-member consortium developed through isolation from healthy mouse feces, was shown to ameliorate the degraded intestinal barrier function of mice with dextran sodium sulfate (DSS)-induced colitis showing significantly improved clinically-relevant outcomes, such as reduced weight loss, decreased Disease Activity Index (DAI), and higher survival rate.^51^ BCT appears to function by inducing the upregulation of the tight junction protein occludin via a mechanism involving the expansion of IL-17A-secreting γδ T cells.

Similarly, a simplified human intestinal microbiota (SIHUMI) community has been studied in an interleukin−10-deficient (IL10^-/-^) mouse model of spontaneous colitis.^53^ SIHUMI is a 7-member community designed through the selection of 7 common microbes found in the human gut microbiota.^52^ Ring et al. used SIHUMI to mimic the human microbiome to investigate the role of *Akkermansia muciniphilia* BAA−835 in promoting colitogenesis in the context of complex microbiota. While *A. muciniphilia* is generally associated with health, it displays strain-level variation, exacerbating gut inflammatory responses during *S.* Typhimurium infection.^95^ When germ-free IL10^-/-^ mice were colonized with SIHUMI and *A. muciniphilia* BAA−835, their ceca presented with adverse histological changes compared to uncolonized IL10^-/-^ mice, but their colons did not show any signs of inflammation. SIHUMI and *A. muciniphilia* BAA−835 also prevented the increase in the transcription of inflammatory markers, including TNFɑ, IFNγ, and Reg3γ, all of which were increased in mice colonized with the colitogenic strain *E. coli* NC101. While the mechanistic driver behind immune modulation by SIHUMI remains unknown, the authors hypothesized that two of its members, *Bacteroides thetaiotaomicron* and *Bifidobacterium longum,* which have been shown to induce host genes involved in immunoinflammatory responses, are responsible.

One of the proposed mechanisms for microbial influence of the host immune response is through production of short-chain fatty acids (SCFAs), which are fermentation products that mediate immune cell proliferation, differentiation, apoptosis, and gut barrier integrity.^96-98^ Some SCFAs, such as butyrate and propionate, have demonstrated protective effects against immune disorders.^99^^,^^100^ To determine whether a defined consortium could impart therapeutic benefits to IBD patients, Ambat et al. designed SC−4, which contains four low-abundance strains with the capacity to produce butyrate. After germ-free mice were stably colonized, SC−4 produced SCFAs modulating the host immune system, significantly decreasing the number of CD8^+^ cytotoxic T cells and increasing the number of CD4^+^ helper T cells and RORγt^+^ T_reg_ cells. SC−4 also conferred protection against DSS-induced colitis, increasing the colon length and improving the DAI score. Surprisingly, three of the four strains in SC−4 could engraft in mice colonized with a complex human microbiome (humanized) and improve the disease outcome after DSS administration.

Further preclinical development of defined communities for IBD has focused on bottom-up approaches to synthesize key metabolites implicated in intestinal mucosal homeostasis, including propionate and indole compounds, to modulate host immune functions and control pathobionts enriched in IBD patients.^56^ GUT−103 was designed from 17 strains to capture desired functions and form a stable consortium. GUT−103 was tested for its efficacy in an IL10^-/-^ colitis model in which colitis was induced via inoculation with *Escherichia coli*,*Enterococcus faecalis*, and *Ruminococcus gnavus* (EER). GUT−103 was demonstrated to effectively colonize the gut and reduce the expression of IFNγ, IL-12p40, and TNFɑ, regardless of preventative (before ERR) or therapeutic (after EER) administration. After validation of efficacy, GUT−103 was refined to the 11-member GUT−108 by removing strains with redundant functions. GUT−108 displayed comparable establishment in the gut of germ-free IL10^-/-^ mice and modulation of host immune function while also reducing inflammatory cytokine expression in humanized IL10^-/-^ mice with induced colitis, showing its potential for clinical application.

Synthetic communities have also been leveraged to understand the role of gut microbes in mediating food allergy (FA). Germ-free mice have reduced gut immunoglobulin A (IgA), T_reg_ cells and a reduced capacity to develop oral tolerance to innocuous antigens.^101^^,^^102^ When the microbiota of infants with FA and age-matched controls are compared, Abdel-Gadir et al. found significant deficiency in Clostridia clusters I, IV, XI, and XIVa in the FA group.^54^ FMT from control patients to germ-free *Il4ra*F709 mice, which are genetically prone to FA, followed by OVA challenge led to a decrease in OVA-specific IgE, but FMT using the samples from the FA group failed to confer the same protection from food allergy with increased markers of anaphylaxis. Using a defined consortium of six Clostridiales type strains deficient in FA patients, the authors showed increased tolerance to oral OVA administration when the strains were colonized in both germ-free and conventional *Il4ra*F709 mice, rescuing the host from the FA phenotype. A second defined consortium of five immunomodulatory Bacteroidales species of human origin was also found to be protective against FA. Strikingly, both the Clostridiales and Bacteroidales consortia were also able to suppress FA in mice with established OVA sensitivity. The mechanism of action was attributed to the activation of RORγt^+^ T_reg_ cells. These findings underscore the significant clinical importance of bacteria-mediated immune regulation in the prevention and treatment of food allergies.

##### Microbes in cancer development and therapy

Zhou et al. developed a defined microbial consortium that can effectively exclude *Fusobacterium nucleatum* and alleviate colorectal cancer (CRC) through a model-based approach.^61^ *F. nucleatum* is a microbe that has been demonstrated to directly contribute to carcinogenesis.^103^ Using a model-based design approach with metabolic networking, they designed SynCom, a 7-member community with complementary metabolic capacity. They were able to bypass the labor-intensive iterative colonization and isolation process by using *in silico* tools, such as Metage2Metabo and pathway tools, to identify the strains that can utilize most amino acids, bile acids, and sugars and whose safety has already been experimentally verified. As a community, SynCom was hypothesized to outcompete *F. nucleatum* for the nutritional substrates found in its niche. After successful colonization and safety were verified in germ-free mice, SynComm, and *F. nucleatum* were administered to mice treated with azoxymethane and DSS to induce CRC. Mice gavaged with only *F. nucleatum* displayed increased blood in the stool, decreased weight, and high DAI scores, but all symptoms were ameliorated with SynCom treatment. Compared with *F*. nucleatum**-only mice, SynCom administration also increased colon length and decreased tumor number and spleen weight*.* While *F. nucleatum* promotes CRC proliferation through the IL−8/TNFα pathway, SynCom downregulates both circulating IL−8 and TNFα levels.

Tanoue et al. designed an 11-member community to induce IFNγ^+^ CD8^+^ T cells in the intestine and enhance antitumor immunity by leveraging principles of iterative experimental design.^55^ They colonized germ-free mice with fecal samples from healthy donors and selected recipients with the strongest induction of IFNγ^+^ CD8^+^ T cells. They gavaged the cecal contents of these recipients into additional germ-free mice and treated them with various antibiotics. Most antibiotic treatments abolished the induction of IFNγ^+^ CD8^+^ T cells, but ampicillin-treated mice retained this ability. From those mice, 26 unique strains were isolated, 21 of which were only found in mice treated with ampicillin. Through additional colonization experiments, they determined that 11 of 21 strains were responsible for the induction of IFNγ^+^ CD8^+^ T cells while the remaining 10 strains (10-mix) were incapable of exhibiting any induction. The 11-strain community (11-mix) was administered to germ-free mice that were subcutaneously engrafted with MC38 adenocarcinoma cells and compared with germ-free or 10-mix-colonized mice. Mice in the 11-mix community had a greater response to anti-PD−1 therapy, an immune checkpoint inhibitor (ICI) used in cancer treatment, and had an increased frequency of IFNγ^+^ CD8^+^ tumor-infiltrating lymphocytes (TILs). In addition, 11-mix significantly suppressed tumor growth even in the absence of anti-PD−1 treatment, suggesting that the 11-mix community is effective in both spontaneous and ICI-mediated antitumor immunity in a CD8^+^ T cell-dependent manner. Importantly, the mice colonized with the 11-mix and treated with anti-PD−1 therapy presented no histological evidence of colitis, which is a common side effect of ICI therapies.

### Synthetic communities in the clinic

In parallel with the basic science research that has demonstrated the remarkable potential of synthetic microbial communities, these communities have also been progressing through the clinical translational pipeline of live therapeutic products (LBPs). While most early applications of FMT focused on CDI, experimental applications of FMT have increased greatly. A current query for studies involving FMT at clinicaltrials.gov reveals hundreds of registrations for studies examining indications including but not limited to aging, alopecia, anorexia, autism, cirrhosis, colorectal cancer, depression, IBD, insomnia, irritable bowel syndrome, and weight loss. Risk-benefit analysis of conventional FMT for each of these indications must be carefully considered. However, much new research and development has shifted the focus to synthetic communities or defined LBPs.^104^ Here, we summarize the current regulatory landscape in the United States and some of the products that are currently approved for market or in clinical trials (Table 2).

**Table 2.** Microbial communities in the clinic.

Name	Company	Number of species	Disease Model	Proposed Mechanism	Clinical Trial Status	Reference, NCT identifier
REBYOTA/RBX2660	Rebiotix/Ferring Pharmaceuticals	Undefined	rCDI	Restoration of gut microbiome	FDA-approved	Blount et al., [105] Khanna et al., [106] Dubberke et al., [107] Kwak et al.,[124] Feuerstadt et al., [108] NCT02589847, NCT02299570, NCT03931941, NCT03244644
VOWST/SER−109	Seres Therapeutics	Undefined	rCDI	Nutritional competition and production of modulatory metabolites	FDA-approved	McGovern et al., [109] Feuerstadt et al., [110] NCT02437487, NCT03183141, NCT03183128, NCT02437500
Xervyteg/Maat013	Maat Pharma	Undefined	acute GvHD	Production of anti-inflammatory SCFAs	Phase III/Early Access Program	NCT03359980, NCT04769895, NCT04768907
			ICI cotherapy		Phase II	NCT04988841
VE303	Vedanta Biosciences	8	rCDI	Nutritional competition and production of modulatory metabolites	Phase III	Menon et al*,*[80] Dsouza et al., [111] Louie et al*,*[112] NCT04236778, NCT03788434, NCT06237452
MET−2	Nubiyota	40	rCDI	Restoration of gut microbiome	Phase I	NCT02865616
			UC		Phase I	NCT03832400
			Depression, Anxiety		Phase II	NCT04052451, NCT04602715
CP−101	Finch	undefined	rCDI	Restoration of gut microbiome	Phase III (discontinued)	NCT03110133, NCT03497806, NCT05153499
SER−287	Seres Therapeutics	undefined	UC	-	Phase II (discontinued)	NCT02618187, NCT03759041
SER−301	Seres Therapeutics	18	UC	Immune modulation	Phase I (discontinued)	ACTRN12620001103954
VE202	Vedanta Biosciences	16	UC	Ecological control, gut barrier restoration, immune modulation	Phase II (discontinued)	Silber et al.,[113] NCT05370885
MB310	Microbiotica	8	UC	Gut barrier restoration, immune modulation	Phase I	NCT06582264
BMC333	Biomica	4	IBD	Immune modulation	Preclinical	Tirosh et al.[114]
MET4	Nubiyotica	30	ICI cotherapy	Immune modulation	Phase I	Spreafico et al., [115] NCT03686202
MB097	Microbiotica	9	ICI cotherapy	Immune modulation	Phase I	Robinson et al.,[116] NCT06540391
MaaT034	Maat Pharm	Undefined	ICI cotherapy	Production of anti-inflammatory SCFAs	Preclinical	Laperrousaz et al.[117]
SER−155	Seres Therapeutics	Unreported	infection and acute GvHD in allogeneic HSCT	Colonization resistance	Phase I	NCT06801067, NCT04995653
MaaT033	Maat Pharm	Undefined	infection and acute GvHD in allogeneic HSCT	Production of anti-inflammatory SCFAs	Phase II	NCT04150393
MET−3	Nubiyotica	Unreported	Hypertriglyceridemia	-	Phase I	NCT03660748, NCT04507971

#### Regulatory landscape

Live Biotherapeutic Products (LBPs) are defined as products containing live organisms that are intended to prevent, treat, or cure a disease or condition in humans and are not vaccines.^118^^,^^119^ Currently, in the United States, LBPs are under the purview of the FDA’s Center for Biologics Evaluation and Research (CBER) and follow the Biologics License Application pathway for approval. Unlike conventional probiotics, which are generally classified as dietary supplements or foods, LBPs are treated as medicinal products and must undergo rigorous clinical testing to demonstrate their safety, efficacy, and quality, similar to conventional pharmaceuticals, including phase I, II, and III trials. However, unlike traditional pharmaceutical products such as small molecules or biologics, including antibodies and proteins, LBPs often do not have a single defined target or mechanism of action but efficacy is instead mediated through complex interactions with the host’s commensal microbes, metabolism, and immune system. Furthermore, inherent variability in live microbial products requires specialized manufacturing processes, quality control measures, and stability testing protocols, including strain identification, contamination screening, and maintenance of viability throughout the product life cycle.^118^

However, gaps still remain in the LBP regulatory framework with anticipated ongoing refinement and collaboration with industry to address these challenges. Traditional pharmaceuticals typically undergo toxicology, pharmacology, and immunogenicity assessment to determine the safety of novel compounds or biologics, but LBPs have additional considerations, such as colonization efficacy and persistence, possible adverse interactions with the host microbiome, risk of transmission between individuals, and risk of transmission of antibiotic resistance.^120^^,^^121^ Every bacterial strain in the LBP should be thoroughly characterized with information on identity, origin, and the potential presence of undesirable genes, including virulence factors and antibiotic resistance.^118^ In addition, various new considerations must be made when designing clinical trials for LBPs to assess its efficacy and safety. LBPs may contain strains that are generally deemed safe for a healthy population, but the same strains could lead to adverse outcomes when may be administered to patients with markedly different health states, diets, lifestyles, and genetics, leading to large variations in the host microbiome. Furthermore, the product should not reach systemic circulation, but its activity and metabolism may directly or indirectly impart systemic effects on the host. Therefore, the dose of the product may not correlate with its toxicity and warrants rigorous testing.^122^ These requirements are put in place by regulators to ensure safety and efficacy, but they also create significant barriers to entry and increased cost of LBP development.

#### C. difficile infection

Since RBX2660 (REBYOTA) was approved and entered the market, *C. difficile* infection (rCDI) remains a focus for LBPs. CDI is one of the most common hospital-acquired infections and is associated with approximately 500,000 patients, leading to more than 20,000 deaths annually in the United States.^123^ The first line of defense is treatment with antibiotics such as vancomycin and fidaxomicin to clear *C. difficile* and relieve the symptoms of colonic inflammation and debilitating diarrhea. However, the same treatment also represents the main risk factor for CDI, as antibiotics are unable to kill *C. difficile* spores, which germinate after treatment discontinuation and cause recurrent infections. As shown with RBX2660^105^^,^^106^^,^^108^^,^^124^ and in basic research,^17^^,^^26^^,^^125^ microbiome-based therapies provide an effective alternative to repeated administration of antibiotics to combat rCDI through ecological mechanisms.

SER−109 (VOWST) became the first oral microbiome therapeutic to be approved by the FDA in 2023 for prevention of rCDI in adults following standard-of-care (SOC) antibiotic treatment.^110^ Using the fecal-deviation design principle, it is comprised of enriched and purified Bacillota (Firmicutes) spores, which survive the gastric environment and germinate in the intestinal tract. Once engrafted, it can compete for nutrients and modulate bile acids to prevent the outgrowth of *C. difficile*. Following a comprehensive donor screening and subsequent monitoring for disease and pathogen presence, the stool samples undergo ethanol-based purification to inactivate and remove vegetative bacteria, fungi, parasites, and viruses. The product is validated by challenge studies using model organisms of various pathogens to confirm successful exclusion of potential disease-causing organisms and to conform to microbiological purity standards.^126^ The efficacy and safety of SER−109 were evaluated through multiple randomized, placebo-controlled clinical trials.^109^^,^^110^^,^^127^^,^^128^ In demonstration of its safety, patients across all clinical trials who received at least one dose of SER-109 sufficiently tolerated the treatment, requiring mostly mild to moderate interventions in treatment-emergent adverse events (TEAEs) that arose.^109^ In the phase 3 ECOSPOR-III trial of 182 participants with three or more rCDI episodes, patients who received SER−109 showed signs of rapid and robust engraftment of the administered species and significantly outperformed the placebo group, who received only SOC antibiotics to prevent recurrence, regardless of age, antibiotics received, or Charlson comorbidity score.^127^ Engraftment of SER−109 was shown to lead to changes in microbe-associated metabolites, including a reduction in primary bile acids that promote *C. difficile* spore germination and the production of secondary bile acids that inhibit *C. difficile* vegetative growth.^110^^,^^109^

VE303, developed by Vedanta Biosciences, is currently undergoing phase III clinical trials.^80^ VE303 is a defined synthetic community composed of 8 commensal strains in the Clostridia class in clusters IV, XIVa, and XVII. It was developed via a similar approach to the 17-mix for IBD and 11-mix for cancer immunotherapy by Atarashi et al., ^48^ and Tanoue et al., ^55^ respectively, in which donor stool samples were iteratively colonized in a mouse model and isolated to identify the strains effective against the disease. Once the candidates for VE303 were identified, they were tested in mice treated with cefoperazone and infected with *C. difficile* spores, a common rCDI model in rodents.^111^ VE303 substantially increased the survival rate of rCDI mice, from 20% to 75%, and matched the level of protection exerted by another more complex 56-strain consortium and raw human fecal material. It could also significantly suppress the growth of *C. difficile in vitro*. Due to the strong evidence of efficacy of VE303 in combatting CDI in preclinical studies, it advanced into clinical development. In an in-human phase 1 trial, VE303 was administered to 39 healthy volunteer adults with and without vancomycin pretreatment for 5 days to assess dose response, safety, and tolerability.^111^ VE303 was found to be well tolerated, and no serious or severe adverse events (SAEs) related to the dose or duration of VE303 treatment were observed. Strains in VE303, especially with vancomycin pretreatment and multiday dosing, could rapidly colonize the gut, with most strains detected in stool within 24−48 hours of administration.^80^^,^^111^ VE303 led to distinguishable metabolic signatures such as increased concentrations of secondary bile acids and short-chain fatty acids.^111^ In phase II, VE303 was tested against placebo control in 79 patients with at least one confirmed CDI episode to test its efficacy in preventing rCDI.^80^^,^^112^ High doses of VE303 led to successful and rapid colonization of 5−8 of its members and had a significantly lower rate of recurrence, 13.8% compared to 45.5% in the placebo group, representing a greater than 80% reduction in the odds of recurrence.^112^ While most patients experienced at least one TEAE, they were mild to moderate, mirroring the results of the SER−109 trial.^112^

#### Inflammatory bowel disease

Inflammable bowel disease (IBD) is a chronic immune-mediated disease that is predominantly composed of two forms that differ in localization: Crohn’s disease (CD) and ulcerative colitis (UC).^129^^,^^130^ Although IBD has traditionally been regarded as a Western disease, its global incidence is rapidly accelerating as newly industrialized countries become more westernized.^131^^,^^132^ IBD is estimated to affect over 0.3% of the global population, and its prevalence is estimated to reach 2% in some parts of the world by 2040, levying a heavy burden on the global healthcare system though both direct and indirect costs.^132-135^ Current clinical interventions primarily focus on controlling inflammation through the use of anti-inflammatory and immunosuppressive drugs. However, LBPs are gaining traction as next-generation therapies to combat IBD, as a growing body of genetic associations and animal studies link the microbiome to its pathogenesis.^136^^,^^137^

SER−287, developed by Seres Therapeutics, is a purified spore preparation to treat patients with ulcerative colitis similar to SER−109. The goal of SER−287 was to reconstitute Bacillota bacteria that can produce metabolites implicated in the maintenance of the intestinal barrier and mucosal immunity, and are reduced in the microbiota of patients with UC.^138^^,^^139^ While promising results were shown in preclinical testing and phase I trials^139^ revealing robust clinical remission compared to placebo and safe engraftment, it ultimately failed in phase II clinical trials and was unable to improve clinical remission rates compared to placebo in patients with mild-to-moderate UC.^140^ However, another Seres product, SER−301, a defined microbial community of a consortium of 18 strains, has shown positive preclinical testing with animal models, reporting robust modulation of colonic CD4^+^ T cells in DSS-induced colitis mice and attenuation of intestinal inflammation in IL10^-/-^ mice.^141^

Leveraging the results described earlier by Atarashi et al. current efforts are underway to develop VE202 for IBD indications, a defined community of 16 strains.^48^^,^^113^ Its proposed mechanism of action is three-pronged: resolution of gut dysbiosis and reduction of pro-inflammatory Enterobacteriaceae, restoration of gut barrier integrity, and modulation of the balance of T_reg_ cells and T_H_17 cells to combat inflammation.^142^ In its phase I trial, VE202 was administered to 31 healthy adults following vancomycin pretreatment, and it has been demonstrated that it is well tolerated with no dose-dependent pattern of adverse effects.^113^ VE202 was able to provide robust colonization of the administered strains, which remained above baseline 24 weeks after initial dosing.^113^ It underwent a double-blind, placebo-controlled randomized phase II clinical trial with patients who fail to reach remission with SOC anti-inflammatory drugs.^143^ Unfortunately, it was unable to outperform placebo in its clinical response and failed to meet the primary efficacy endpoint resulting in the phase II clinical trial being terminated.

#### Immune checkpoint inhibitor therapy

Anti-PD−1 immunotherapy has emerged as a breakthrough cancer therapy that functions to block programmed cell death protein 1 (PD−1) on the surface of CD8^+^ T cells or its partner ligand PD-L1 on tumor cells, allowing cytotoxic T cells to sustain antitumor activity.^144^^,^^145^ However, anti-PD−1 therapy has benefited only a subset of patients, and one of the variables contributing to interpatient heterogeneity has been attributed to the gut microbiome.^146^ Some commensal species, such as *Bifidobacterium longum*, *Collinsella aerofaciens*, and *Enterococcus faecium*, are significantly enriched in therapeutic responders, and *A. muciniphila* was found to be deficient in nonresponders.^147-149^ Furthermore, FMT from healthy donors has been shown to improve treatment outcomes.^150-153^

Informed by the RePOOPulate and related works, Nubiyota developed Microbial Ecosystem Therapeutic 4 (MET4), a defined consortium of 30 strains that includes taxa associated with ICI responsiveness.^115^ MET4 completed a phase I trial to assess safety, tolerability, and ecological response in patients with solid malignant tumors in combination with ICI treatment.^115^^,^^154^ Consistent with other defined communities, MET4 was found to be safe and well tolerated compared to control subjects receiving only anti-PD−1 monotherapy or anti-PD−1/anti-CTLA−4 combination therapy, and mild-to-moderate adverse events occurred in 17% of patients. Patients who received MET4 showed significant engraftment of some, but not all, species in the consortium. Importantly, taxa associated with an increased ICI response, such as *Bifidobacterium* and *Enterococcus,* were detected at relatively high levels in the MET4 recipients. Furthermore, increases in circulating anti-MET4 immunoglobulin G (IgG), B cells, and FOXP3^+^ and CD4^+^ T cells were detected in MET4 recipients via flow cytometry, indicating the induction of treatment-specific humoral immunity by the bacterial consortium.^154^ However, while MET4 increased the ICI response rate compared to control, the difference was not statistically significant, so the clinical benefits of MET4 supplementation were deemed inconclusive.

Microbiotica developed MB097 to specifically target melanoma patients receiving anti-PD−1 therapy.^116^ MB097 is a defined community of 9 species that are significantly associated with ICI efficacy across multiple cancer studies, including the Cambridge MELRESIST study, which included a cohort of advanced melanoma patients receiving ICI treatments. Preclinical *in vitro* and *in vivo* studies demonstrated that MB097 stimulates dendritic cells to enhance the CD8^+^ T cell response and promote antigen presentation. A phase 1B trial is currently underway to investigate the safety, tolerability, and efficacy of MB097 in combination with pembrolizumab for anti-PD−1 therapy in patients with advanced melanoma.^155^

## Conclusions and future perspective

Microbiome sciences have grown exponentially in recent decades bringing greater understanding of the role that commensal microbes play in shaping human health. Synthetic microbial communities have repeatedly demonstrated their capacity as powerful research tools and innovative new therapies. However, to develop effective therapeutic strategies, understanding the causal mechanisms behind how microbes shape health and disease is paramount. Just as successful small-molecule or biological pharmaceuticals depend on mapping receptor‒ligand interactions and their downstream cascades,^156^^,^^157^ microbiome therapeutics require the identification of specific organisms and their functional contributions to physiological, metabolic, and immunological outcomes. Synthetic community studies have begun to illuminate these microbial players and their interactions, but the transition from empirical microbiome research to rationally designed, mechanistically informed therapeutics that reliably restore health across patient populations will require addressing the gaps in the understanding of community assembly rules, metabolic cross-feeding networks, and host–microbe signaling pathways that govern therapeutic efficacy.

Clinical successes in the treatment of recurrent *C. difficile* infection have validated the regulatory pathway for LBPs, while the economic viability of these approaches remains to be determined. While challenges still remain in translating the research findings and preclinical candidates to validated therapeutic LBPs, the lessons learned from first-generation products will transform the treatment landscape for a broad spectrum of complex human diseases. By integrating novel mechanistic insights from research on defined microbial consortia with evolving biomarker-guided patient stratification through advances in analytical techniques, microbiome-based therapeutics are positioned to transition from microbial restoration via FMT to precision medicine that is tailored to different therapeutic needs across gastroenterology, immunology, and cancer biology.

## Data Availability

No new data were generated as a component of this manuscript.

## Abbreviations

FMT: Fecal microbiota transplant
CDI: *Clostridioides difficile* infection
rCDI: Recurrent *Clostridioides difficile* infection
SAE: Severe adverse effect
LBP: Live biotherapeutic product
GF: Germ-free
SF: Schaedler flora
ASF: altered Schaedler flora
SPF: Specific pathogen-free
IBD: Inflammatory bowel disease
DSS: Dextran sodium sulfate
DAI: Disease activity index
IL: Interleukin
FA: Food allergy
IgA: Immunoglobulin A
IgG: Immunoglobulin G
SCFA: Short-chain fatty acid
HC: Healthy control
OVA: Ovalbumin
TNBS: Trinitrobenzenesulfonic acid
IFNγ: Interferon gamma
ICI: Immune checkpoint inhibitor
T_reg_: Regulatory T cells
CRC: Colorectal cancer
FDA: Food and Drug Administration
SOC: Standard-of-care
TEAE: Treatment-emergent adverse event
UC: Ulcerative colitis
CD: Crohn’s diseases
EER: *Escherichia coli*, *Enterococcus faecalis*, and *Ruminococcus gnavus*
IACR: International Association for Cancer Registries
PD−1: Programmed cell death protein 1
